# Treatment Patterns of Follicular Lymphoma in the United States: A Claims Analysis

**DOI:** 10.36469/001c.38070

**Published:** 2022-10-24

**Authors:** Scott F. Huntington, Sreevalsa Appukkuttan, Wenyi Wang, Yuxian Du, Sari Hopson, Svetlana Babajanyan

**Affiliations:** 1 Yale University, New Haven, Connecticut; 2 Bayer Healthcare U.S. LLC, Whippany, New Jersey; 3 Xcenda, Carrollton, Texas

**Keywords:** follicular lymphoma, treatment patterns, relapsed/refractory treatment regimen, claims analysis

## Abstract

**Background:** A consensus is lacking on optimal treatment sequencing for follicular lymphoma (FL), the most common indolent lymphoma. FL is incurable, and many patients require multiple lines of therapy for successive relapses. Guidelines provide numerous recommendations for first-, second-, and third-line therapy; however, treatment patterns in the real world remain poorly understood.

**Objectives:** The primary objective of this study is to evaluate real-world treatment patterns among commercially insured patients with FL in the United States.

**Methods:** A retrospective cohort of patients with newly diagnosed FL was identified from June 2008 to September 2016 using the IBM MarketScan® database. Treatment pattern measures, including time to treatment from diagnosis, days from previous line of therapy, duration of therapy, and distribution of treatment regimens among lines of therapy, were assessed. Descriptive statistics were reported for baseline characteristics, primary outcome, and treatment pattern measures.

**Results:** In total, 4232 patients were identified from the database and 2111 patients received at least 1 line of treatment. The most common first-line treatments included bendamustine + rituximab (39%), rituximab + cyclophosphamide + doxorubicin + vincristine (20%), and rituximab monotherapy (19%). Rituximab monotherapy was the most common second-line (34%) and third or greater line (57%) treatment. The median time from FL diagnosis to initiation of treatment was 50 days (interquartile range [IQR]: 28-191) for first-line treatment, 577 days (IQR: 312-1146) for second-line, and 776 days (IQR: 603-1290) for third-line.

**Discussion:** At a median follow-up of 3.6 years, most patients had 1 or fewer lines of therapy. The use of combination therapy decreased with each line of therapy and the numbers of patients receiving third- or fourth-line therapy were small in this study, potentially due to the short follow-up. Rituximab as monotherapy or in combination was utilized most frequently; however, the variety of other therapies used demonstrates that the standard management of FL remains unclear.

**Conclusions:** Consensus on optimal treatment sequencing is currently lacking, and patients receive a variety of active regimens during routine practice. In this contemporary cohort of patients diagnosed with FL in the United States, rituximab therapy predominated both in monotherapy and in combination.

## INTRODUCTION

Follicular lymphoma (FL) is the second most common lymphoma and the most common indolent lymphoma diagnosed in the United States and Western Europe, with around 14 000 cases diagnosed in the United States annually.^1,2^ FL represents 20% to 35% of all non-Hodgkin lymphomas (NHLs) and 70% of indolent lymphomas.^3–5^ Although FL is incurable, it most frequently follows an indolent waxing and waning course.^6^ As a result, it is necessary to have a wide variety of treatment options to deal with successive relapses.^7^ The goal of FL-directed therapy is to establish a quality remission that is durable and without excessive toxicity, though treatment is far from standardized.

Despite considerable improvements in available therapies for FL, consensus is lacking on optimal treatment sequencing. Many guidelines and studies provide recommendations for first-line therapy, second-line therapy, and subsequent use; however, treatment selection is left for clinicians and patients to make based on a variety of criteria.^8–11^ The real-world sequencing of treatments for FL remains poorly understood. This is attributed to the fact that there are limited publications describing the treatment sequences used in affected patients in the United States.^12–20^ These studies used data from a variety of sources including the National LymphoCare Study,^12,13,16^ commercial health plans, Medicare, and electronic medical records to provide important information on treatment patterns and outcomes; however, they did not capture utilization of newer agents, such as idelalisib, ibrutinib, or obinutuzumab.

A retrospective study among FL patients identified 598 patients through the IBM MarketScan® database between January 1, 2010, and December 31, 2013, who received at least 1 line of treatment for FL.^19^ In that patient population, rituximab was utilized as monotherapy or in combination therapy most frequently across all lines of treatment, and the observed treatment patterns conformed with National Comprehensive Cancer Network recommendations. This current study builds upon those results by leveraging a larger sample size and adds to previous literature by including newer agents used in contemporary practice.

Conducting an FL real-world treatment patterns study that captures newer agents is important to inform stakeholders, understand the burden of disease, and guide future research. The primary objective of this study is to describe contemporary real-world treatment patterns, including treatment sequences, types, and durations for patients with FL who are commercially insured in the United States.

## METHODS

### Study Design and Patient Population

This retrospective observational study analyzed IBM MarketScan® Commercial Claims and Encounters and Medicare Supplemental and Coordination of Benefits databases. MarketScan® claims databases contain healthcare data for more than 43.6 million covered lives and are large enough to allow creation of a nationally representative data sample of Americans with employer-sponsored health insurance. The database includes enrollment history and claims for medical services (provider and institutional), pharmacy services, and standard demographic variables and clinical characteristics.^21^ Patients with 1 or more FL diagnosis between October 1, 2008, and September 30, 2016 (identification [index] period), were selected. Because FL did not have a specific *International Classification of Diseases* (ICD)*, Ninth Revision* (ICD-9) code, we required patients to have claims beyond the start date of the *ICD*, *Tenth Revision* (ICD-10) (October 1, 2015) with an ICD-10 code specific for FL (C82.xx). We then evaluated for earlier FL diagnosis in these patients for an ICD-9 code corresponding to indolent NHL. The earliest observed date of FL or an indolent NHL diagnosis served as the index date. The baseline period was the 12-month period prior to each eligible patient’s index date (pre-index period). The study observation (post-index) period started from the index date and continued until the earliest of the last day of continuous enrollment or the end of the study.

### Study Variables and Outcomes

The following primary outcomes regarding treatment patterns were described within each line of therapy: time to treatment from diagnosis, days from previous line of therapy, and duration of therapy. In the post-index period, distribution of treatment types and regimens among lines of therapy (eg, first, second, third, fourth) and time to treatment (days) and duration (days) of treatment for each line of therapy were assessed. Time to treatment was measured as the days from diagnosis to first-line therapy and the days from previous line of therapy to subsequent line of therapy. Duration of treatment was measured as the days from start of line of treatment to end of line of treatment (eg, start of line of therapy 1 [days] to end of line of therapy 1 [days], start of line of therapy 2 [days] to end of line of therapy 2 [days]).

### Measurement of Lines of Therapy

First-line therapy was considered as the first treatment type or regimen that the patient received any time after the diagnosis. Each subsequent line of therapy was defined by the start of a different treatment or regimen, except for maintenance rituximab or maintenance obinutuzumab. Line of therapy was defined by treatment type/regimen, start date, end date, duration, and the sequence in which treatments are given after diagnosis. The start of each line of therapy was defined as the first prescription fill or service date for the first injection. The end of each line of therapy was determined as the date of last prescription fill and adding the days’ supply or the service date of last injection, prior to (1) a gap of more than 60 days, except for rituximab monotherapy plus maintenance, where the allowable gap prior to discontinuation was up to 200 days; (2) starting a new treatment type/regimen; or (3) loss to follow-up or end of study period.

Combination therapies included bendamustine + rituximab (R); bendamustine + R + rituximab maintenance (RM); lenalidomide + R; idelalisib + R; yttrium + R; rituximab + cyclophosphamide + doxorubicin + vincristine (R-CHOP); R-CHOP + RM; R-cyclophosphamide + vincristine + prednisone (CVP); R-CVP + RM; bendamustine + obinutuzumab; cyclophosphamide + vincristine + doxorubicin (CHOP) + CVP. Maintenance rituximab and maintenance obinutuzumab are exclusions that were not considered a separate, unique line of therapy. Maintenance rituximab or obinutuzumab were identified as injections that were spaced at least 7 weeks apart and occurring after a previous treatment type. Other procedures that were reported but not counted as a line of therapy were autologous stem cell rescue (Current Procedural Terminology [CPT] code 38241) and allogeneic hematopoietic cell transplant (CPT code 38240).

### Statistical Analysis

Descriptive statistics for baseline demographic, clinical, and comorbid characteristics and primary outcome and treatment pattern measures were reported. Descriptive statistics include means, SD, 95% confidence intervals (CIs), medians, and interquartile ranges (IQRs) for continuous variables. Frequencies and proportions were reported for categorical variables.

## RESULTS

### Patient Attrition

**Figure 1.**
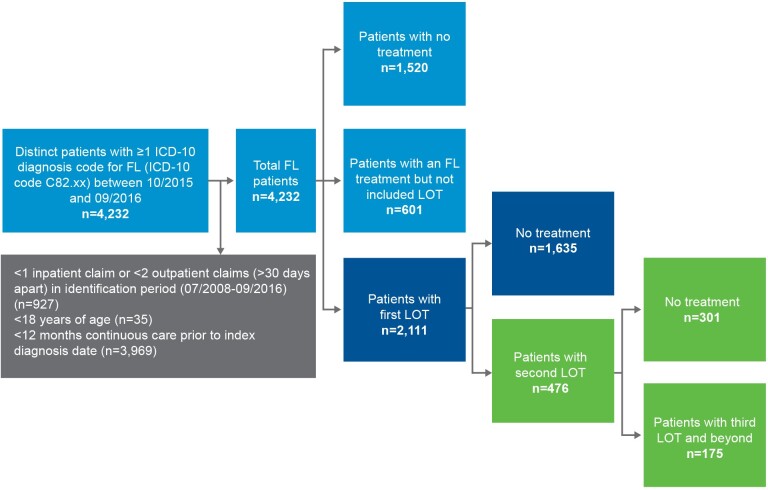
Sample Attrition Abbreviations: FL, follicular lymphoma; ICD, *International Classification of Diseases*; LOT, line of treatment.

A total of 4232 distinct patients with at least 1 ICD-10 diagnosis code for FL (ICD-10 code C82.xx) between October 1, 2015, and September 30, 2016, were identified from the IBM MarketScan**®** database and included in the study (Figure 1). Of these, 2111 patients had at least 1 listed line of treatment for FL (excluding dexamethasone and prednisone) during a median of 2.2 (mean, 3.59; SD, 2.2) years of follow-up. Among those who received a first-line treatment, 476 had a second line of FL-directed treatment over an average 2.71 (SD: 2.1) years of follow-up. Further, 175 patients had a third line of FL-directed treatment for FL over an average of 2.30 years of follow-up.

### Baseline Characteristics

The median follow-up across all 4232 patients was 1030.5 days (mean, 1227; SD, 823) and was 1176.5 days (mean, 1354; SD, 809) among the treated cohort of 2712. Treated patients had a median age of 61 years and 53% were male (Table 1). The majority of treated patients had employer insurance (62.8%) compared with Medicare Advantage (37.2%).

**Table 1.** Baseline Characteristics

**Characteristic**	**Treated (n = 2712)**	**Untreated (n = 1520)**
Median follow-up time	1176.5 days (3.2 y); SD: 809 days (2.2 y)	1030.5 days (2.8 y); SD: 823 days (2.6 y)
Age (y), mean (SD)	61.6 (12.7)	62.5 (12.9)
Age group, n (%)		
18-34	39 (1.4)	35 (2.3)
35-44	184 (6.8)	78 (5.1)
45-54	551 (20.3)	264 (17.4)
55-64	922 (34.0)	529 (34.8)
65-74	548 (20.2)	325 (21.4)
≥75	468 (17.3)	289 (19.0)
Gender, n (%)		
Female	1285 (47.4)	736 (48.4)
Male	1427 (52.6)	784 (51.6)
Geographic region, n (%)		
Northeast region	451 (16.6)	418 (27.5)
North central region	897 (33.1)	409 (26.9)
South region	1008 (37.2)	463 (30.5)
West region	354 (13.1)	228 (15.0)
Unknown region	2 (0.1)	2 (0.1)
Health plan, n (%)		
Commercial	1703 (62.8)	918 (60.4)
Medicare	1009 (37.2)	602 (39.6)
Year of diagnosis, n (%)		
2008	69 (2.5)	29 (1.9)
2009	151 (5.6)	102 (6.7)
2010	169 (6.2)	75 (4.9)
2011	204 (7.5)	83 (5.5)
2012	298 (11.0)	160 (10.5)
2013	363 (13.4)	196 (12.9)
2014	478 (17.6)	238 (15.7)
2015	589 (21.7)	373 (24.5)
2016	391 (14.4)	264 (17.4)
Baseline comorbidity, n (%)		
Myocardial infarction	50 (1.8)	32 (2.1)
Congestive heart failure	112 (4.1)	83 (5.5)
Peripheral vascular disease	199 (7.3)	123 (8.1)
Cerebrovascular disease	180 (6.6)	114 (7.5)
Dementia	7 (0.3)	5 (0.3)
Chronic pulmonary disease	416 (15.3)	216 (14.2)
Connective tissue disease, rheumatic disease	109 (4.0)	39 (2.6)
Peptic ulcer disease	41 (1.5)	15 (1.0)
Mild liver disease	218 (8.0)	106 (7.0)
Diabetes without complications	483 (17.8)	264 (17.4)
Diabetes with complications	96 (3.5)	58 (3.8)
Paraplegia and hemiplegia	7 (0.3)	15 (1.0)
Renal disease	137 (5.1)	81 (5.3)
Cancer	716 (26.4)	407 (26.8)
Moderate or severe liver disease	8 (0.3)	4 (0.3)
Metastatic carcinoma	1 (0.0)	0 (0.0)

### Treatment Patterns

**Figure 2.**
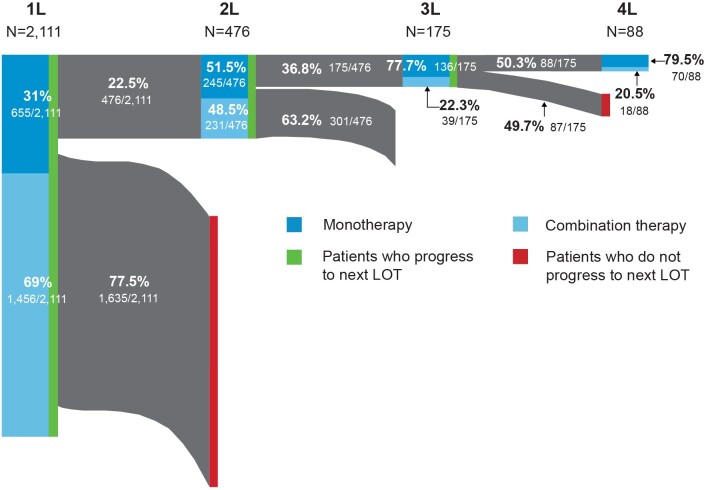
Treatment Patterns by Line of Therapy Abbreviation: LOT, line of treatment.

**Figure 3.**
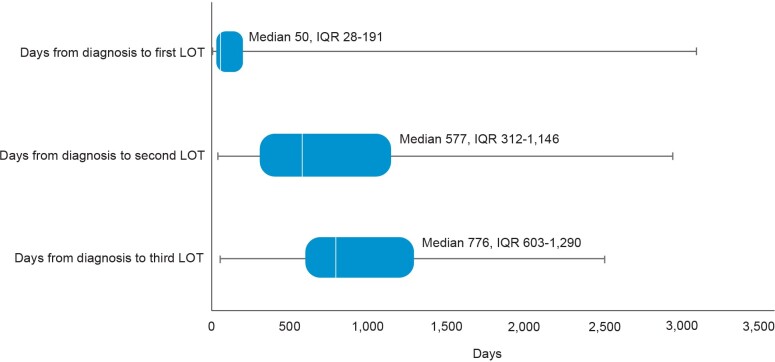
Time (Days) From Diagnosis to Line of Treatment Abbreviations: IQR, interquartile range; LOT, line of treatment.

The distribution of FL-directed treatment categories received and average duration of treatment are presented in Table 2 and Table 3, respectively. Treatment patterns by line of therapy are illustrated in Figure 2. Time from diagnosis for each line of treatment is presented in Figure 3. Only 1 line of treatment was received in 2111 patients, and the median duration of the therapy was 112 days.

**Table 2.** Treatment Patterns by Regimen and Line of Therapy

**Regimen/Treatment**		**Line of Treatment**		
	**First, n (%)**	**Second, n (%)**	**Third, n (%)**	**Fourth, n (%)**
**All treatments**	**2111 (100)**	**476 (100)**	**175 (100)**	**88 (100)**
Monotherapy	655 (31.0)	245 (51.5)	136 (77.7)	70 (79.5)
Combination therapy^a^	1456 (69.0)	231 (48.5)	39 (22.3)	18 (20.5)
Rituximab-based	2031 (96.2)	411 (86.3)	141 (80.6)	83 (94.3)
B-R	830 (39.3)	129 (27.1)	14 (8)	9 (10.2)
B-R + RM	6 (0.3)	—	—	1 (1.1)
Lenalidomide + R	4 (0.2)	7 (1.5)	3 (1.7)	—
Idelalisib + R	—	—	1 (0.6)	—
Yttrium + R	4 (0.2)	4 (0.8)	3 (1.7)	—
R-CHOP	426 (20.2)	30 (6.3)	6 (3.4)	2 (2.3)
R-CHOP + RM	1 (0.0)	—	—	—
R-CVP	160 (7.6)	41 (8.6)	2 (1.1)	3 (3.4)
R-CVP + RM	—	—	1 (0.6)	—
RM	396 (18.8)	160 (33.6)	99 (56.6)	67 (76.1)
R-mono + maintenance	204 (9.7)	40 (8.4)	12 (6.9)	1 (1.1)
Non-rituximab-based	80 (3.8)	65 (13.7)	34 (19.4)	5 (5.7)
Bendamustine + obinutuzumab	4 (0.2)	6 (1.3)	4 (2.3)	2 (2.3)
CHOP	15 (0.7)	11 (2.3)	3 (1.7)	1 (1.1)
CVP	6 (0.3)	3 (0.6)	2 (1.1)	—
Ibrutinib	27 (1.3)	17 (3.6)	8 (4.6)	—
Lenalidomide	24 (1.1)	17 (3.6)	10 (5.7)	—
Idelalisib	4 (0.2)	11 (2.3)	7 (4)	2 (2.3)

Abbreviations: B-R, bendamustine + rituximab; CHOP, cyclophosphamide + vincristine + doxorubicin (either traditional or liposomal) + prednisone; CVP, cyclophosphamide + vincristine + prednisone; R, rituximab; RM, rituximab maintenance; R-mono, rituximab monotherapy.

^a^Combination therapies include bendamustine + R; bendamustine + R + RM; lenalidomide + R; idelalisib + R; yttrium + R; R-CHOP; R-CHOP + RM; R-CVP; R-CVP + RM; bendamustine + obinutuzumab; CHOP + CVP.

**Table 3.** Average Duration of Treatment (Days)

**Regimen/Treatment**	**Line of Treatment**							
	**First**		**Second**		**Third**		**Fourth**	
	**Mean (SD)**	**Median**	**Mean (SD)**	**Median**	**Mean (SD)**	**Median**	**Mean (SD)**	**Median**
Rituximab-based								
R-mono	28.3 (16.4)	21	29.3 (54.6)	21	25.6 (18.8)	21	22.3 (3.1)	21
R-mono + maintenance	483.4 (330.1)	389.5	274.1 (193.8)	199.5	267 (218.1)	170.5	112	112
B-R	119 (48.9)	140	91.9 (58.6)	85	111.6 (57.3)	141	91.7 (56.1)	69
B-R + RM	179.7 (42)	175	—	—	—	—	223	223
Lenalidomide + rituximab	227.5 (74.7)	218	246.4 (225.2)	156	76 (83.1)	28	—	—
Idelalisib + rituximab	—	—	—	—	80	80	—	—
Yttrium + rituximab	7 (0)	7z	7.3 (0.5)	7	7.3 (0.6)	7	—	—
R-CHOP	100.9 (35.2)	112	92.5 (39.9)	115.5	55.8 (34.2)	48.5	49 (29.7)	49
R-CHOP + RM	1 (168)	168	—	—	—	—	—	—
R-CVP	98.8 (38.4)	112	74.3 (49.3)	70	126.5 (9.2)	126.5	89 (53.4)	112
R-CVP + RM	—	—	—	—	266	266	—	—
Non-rituximab-based								
Bendamustine+ obinutuzumab	89.5 (41.1)	109.5	109.2 (50.8)	133	80.5 (63)	70	60 (4.2)	60
CHOP	72.7 (44.2)	70	46.5 (26.3)	28	70.3 (42.5)	70	28	28
CVP	96.5 (55.1)	115	56 (25.2)	63	51 (32.5)	51	—	—
Ibrutinib	355 (281.2)	264	249.4 (343.8)	81	177.3 (186.1)	151	—	—
Lenalidomide	178 (178.2)	135	167.1 (174.9)	77	82.9 (93.1)	55.5	—	—
Idelalisib	117.5 (73.9)	116.5	147.5 (117.9)	117	107.3 (65.8)	142	146.5 (40.3)	146.5
All patients	135.4 (168)	112	94.8 (130.6)	33.5	69.3 (102.6)	24	39.3 (41.4)	21

Abbreviations: B-R, bendamustine + rituximab; CHOP, cyclophosphamide + vincristine + doxorubicin (either traditional or liposomal) + prednisone; CVP, cyclophosphamide + vincristine + prednisone; R, rituximab; RM, rituximab maintenance; R-mono, rituximab monotherapy.

**First-line treatment patterns:** Among first-line treatments, the 3 most common regimens included bendamustine + rituximab (B-R) (39%), R-CHOP (20%), and rituximab monotherapy (R-mono) (19%). Most patients received combination immunochemotherapy (69.0%) over monotherapy (31.0%). The median time from FL diagnosis to initiation of first line of treatment was 50 days (IQR: 28-191); 476 patients received at least 2 lines of treatment. Utilization of the newer agents ibrutinib (1.3%) and idelalisib (0.2%) was low in first-line therapy.

**Second-line treatment patterns**: Among patients who received a second line of treatment, the 3 most common regimens were R-mono (34%), B-R (27%), and R-CVP (9%). Slightly more patients received monotherapy treatment (51.5%) over combination therapy (48.5%), but the proportions were similar. The median time from FL diagnosis to initiation of second line of treatment in our cohort was 577 days (IQR: 312-1146).

**Third-line and beyond treatment patterns**: Among the 175 patients who received a third line of treatment, R-mono was the most common (57%). Less than a quarter (22.3%) of patients received combination therapy. Median time from FL diagnosis to initiation of third line of treatment was 776 days (IQR: 603-1290) and 175 days (IQR: 147-267) from second to third line. Eighty-eight patients had 4 or more lines of treatment. The majority of these patients, 67 (76.1%), received R-mono treatment (nonmaintenance), and only 18 (20.5%) patients received combination therapy. The median duration of the therapy was 21 days. Among those who initiated third-line therapy, 4% initiated idelalisib and 4.6% initiated ibrutinib. Only 2.3% initiated a bendamustine + obinutuzumab regimen.

## DISCUSSION

In a contemporary cohort of commercially insured individuals diagnosed with FL in the US, most patients had 1 or fewer lines of FL-directed therapy at a median follow-up of 3.6 years. Second- and third-line therapies were uncommon in this commercial population, and this may be due to the short follow-up, as most patients with FL will have more than 2 years between the initiation of first- and second-line treatment.^22^ The use of combination therapy regimens decreased with each line of therapy, from 69.0% for the first line of treatment down to 20.5% for the fourth line of treatment. The use of combination therapy as a first-line treatment was slightly higher among patients in our study (69.0%) compared with those reported in a retrospective cohort study of an integrated delivery network (IDN) population (61.4%).^15^ However, the portion of patients receiving combination therapy in later lines of therapy was higher in the IDN population. That study utilized a different population and did not include newer agents, such as idelalisib, ibrutinib, or obinutuzumab, or evaluate beyond the third line of therapy. The greater utilization of monotherapy approaches in later lines of therapy observed in our study may be the result of new agents becoming available.

Similar to previous research, rituximab therapy was frequently utilized across all lines of treatment, with considerable recycling in monotherapy and combination regimens.^13,15,17,19^ However, the frequency of therapies utilized in combination with rituximab varied. In our study, B-R accounted for 39.3% of rituximab-containing first-line regimens. In comparison, an analysis of linked SEER-Medicare data, which characterized treatment patterns among elderly patients with FL who were diagnosed between 2000 and 2013, found B-R accounted for only 13% of rituximab-containing first-line treatment regimens. The findings of our study were more similar to those reported in the IDN population, which showed 43.8% of patients were treated with B-R combination therapy as first line,^15^ along with a recent study using an earlier MarketScan® dataset.^19^ The comparison of our study using a larger and more recent 2008 to 2016 MarketScan® dataset to the recently published 2010 to 2013 MarketScan® analysis identified similar utilization of rituximab among the first 3 lines of therapy. However, it is difficult to draw comparisons between the studies beyond third line of therapy. In the 2010 to 2013 MarketScan® analysis, only 21 patients received fourth-line therapy and 10 patients received fifth-line therapy.

The distribution of patients across a wide range of agents indicates a lack of standard of care in the treatment of FL, which presents challenges in its management in clinical practice. While a broad range of therapies were utilized alone or in combination across each line of therapy, the observed utilization of phosphoinositide 3-kinase inhibitors, such as idelalisib, was low. The utilization of idelalisib was only 0.2%, 2.3%, 4%, and 2.3% from first line to fourth line of treatment. This may be the result of a shorter follow-up time utilized in this study. Some of the patients entered the cohort prior to the novel agents in the market, which may have also contributed to this finding. While this research did not examine the characteristics of early progressors and late progressors, we observed an average of 577 days between diagnosis and second-line therapy for those who progressed to second line during our study, with median follow-up of 3.6 years.

There are limitations that should be considered when interpreting the study results. First, retrospective research conducted with claims data has inherent limitations, including possible coding errors, lack of detailed clinical information, and reasons underlying choice and provision of treatment. It is possible that relevant characteristics were not captured with diagnosis codes, and important clinical information like disease staging is not captured in the claims. Second, patients with FL were identified based on ICD-10 diagnosis codes, and patients’ claims prior to the observed ICD-10 diagnosis code were assessed in the months and years prior to January 1, 2008, for evidence of an earlier diagnosis of FL based on an ICD-9 diagnosis code for indolent NHL or ICD-10 diagnosis code for FL. Prior to the implementation of ICD-10, there was no specific code for FL. We utilized an algorithm to identify indolent NHL patients in the years before 2015. Third, the numbers of patients receiving third- and fourth-line therapy were small in our sample, and the observed treatment patterns might differ in a large sample or a sample accounting for longer follow-up time. Lastly, our short follow-up time limits our ability to capture long-term treatment patterns among this patient population; however, treatment patterns observed early in care were captured among this contemporary cohort.

## CONCLUSION

This large claims database study of FL patients showed that around 1 in 4 patients did not receive FL-directed treatment over a median follow-up of 3.6 years. Among those treated, rituximab therapy predominated both in monotherapy and in combination. The most common treatment regimens were B-R, R-CHOP, and R-mono. Consensus on optimal treatment sequencing is currently lacking, and patients receive a variety of active regimens during routine practice. This analysis provides a unique representation of treatment patterns in a commercial population.

### Author Contributions

S.A. participated in conceptualization, methodology, formal analysis, validation, and writing, reviewing and editing; S. Huntington participated in conceptualization, methodology, validation, and writing, reviewing, and editing; Y.D. participated in conceptualization, methodology, formal analysis, and writing, reviewing, and editing; W.W. participated in conceptualization, methodology, data curation, writing, and reviewing and editing; S. Hopson participated in conceptualization, methodology, data curation, methodology, writing, and reviewing and editing; and S.B. participated in conceptualization, methodology, writing, reviewing and editing, and supervision.

### Disclosures

S. Huntington is a consultant of Bayer, and authors S.A., W.W., Y.D., and S.B. are employees and stockholders of Bayer. Bayer HealthCare Pharmaceuticals, Inc provided funding for this study and oversight on data design, collection, analysis, and interpretation, but the authors maintained intellectual rigor and final approval of the manuscript. Bayer contracted Xcenda to assist in the completion of this study, and at the time of this research, S. Hopson was an employee. Because this was a retrospective analysis that used blinded patient information, IRB approval was not required.

### Presentation

Results from this study were presented as an abstract at the International Society of Pharmacoeconomics and Outcomes Research (ISPOR) 2021 meeting.
